# Heterologous Biosynthesis of the Fungal Sesquiterpene Trichodermol in *Saccharomyces cerevisiae*

**DOI:** 10.3389/fmicb.2018.01773

**Published:** 2018-08-06

**Authors:** Jianghua Liu, Yanan Zhai, Yang Zhang, Shuaiming Zhu, Gang Liu, Yongsheng Che

**Affiliations:** ^1^State Key Laboratory of Mycology, Institute of Microbiology, Chinese Academy of Sciences, Beijing, China; ^2^College of Life Sciences, University of Chinese Academy of Sciences, Beijing, China; ^3^State Key Laboratory of Medicinal Chemical Biology and College of Pharmacy, Nankai University, Tianjin, China; ^4^State Key Laboratory of Toxicology and Medical Countermeasures, Beijing Institute of Pharmacology and Toxicology, Beijing, China

**Keywords:** FPP, heterologous biosynthesis, *Saccharomyces cerevisiae*, trichodiene, trichodermol, RNA-Seq, qPCR

## Abstract

Trichodermol, a fungal sesquiterpene derived from the farnesyl diphosphate pathway, is the biosynthetic precursor for trichodermin, a member of the trichothecene class of fungal toxins produced mainly by the genera of *Trichoderma* and *Fusarium*. Trichodermin is a promising candidate for the development of fungicides and antitumor agents due to its significant antifungal and cytotoxic effects. It can also serve as a scaffold to generate new congeners for structure-activity relationship (SAR) study. We reconstructed the biosynthetic pathway of trichodermol in *Saccharomyces cerevisiae* BY4741, and investigated the effect of produced trichodermol on the host by *de novo* RNA sequencing (RNA-Seq) and quantitative Real-time PCR analyses. Co-expression of pESC::*FgTRI5* using plasmid pLLeu-tHMGR-UPC2.1 led to trichodiene production of 683 μg L^-1^, while integration of only the codon-optimized *FgTRI5* into the chromosome of yeast improved the production to 6,535 μg L^-1^. Subsequent expression of the codon-optimized cytochrome P450 monooxygenase encoding genes, *TaTRI4* and *TaTRI11*, resulted in trichodermol, with an estimated titer of 252 μg L^-1^ at shake flask level. RNA-Seq and qPCR analyses revealed that the produced trichodermol downregulated the expression of the genes involved in ergosterol biosynthesis, but significantly upregulated the expression of *PDR5* related to membrane transport pathway in *S. cerevisiae.* Collectively, we achieved the first heterologous biosynthesis of trichodermol by reconstructing its biosynthetic pathway in yeast, and the reconstructed pathway will serve as a platform to generate trichodermin analogs as potential candidates for agrochemicals and anticancer agents through further optimizations.

## Introduction

Terpenoids are the largest group of natural products mostly isolated from the plants (Sun et al., 2006; Fraga, 2011), but are also frequently encountered as fungal secondary metabolites (Geris and Simpson, 2009). They have been widely used in pharmaceuticals, food additives, and fragrance due to highly diverse structures, physical properties, and biological functions (Chang and Keasling, 2006; Chemler et al., 2006; Ajikumar et al., 2008). Extraction and isolation are the commonly used approaches to obtain pure or mixtures of terpenoids, which are neither environmental friendly nor efficient, while chemical syntheses are still daunting tasks due to their complex structures, rendering metabolic engineering an attractive approach for terpenoid production (Ajikumar et al., 2008; Leonard et al., 2010). Some notable natural products or their precursors including artemisinic acid (Ro et al., 2006), taxadiene (Dejong et al., 2006; Engels et al., 2008; Ajikumar et al., 2010), miltiradiene (Zhou et al., 2012), ginsenoside (Yan et al., 2014), and strictosidine (Brown et al., 2015), have been produced in engineered *Escherichia coli* and *Saccharomyces cerevisiae*. As the largest subgroup of terpenoids with over 7,000 known structures (Sonntag et al., 2015), sesquiterpenes are originated from the common building block farnesyl diphosphate (FPP) generated by condensation of two units of isopentenyl diphosphate (IPP) and a moiety of dimethylallyl diphosphate (DMAPP), both of which are derived from the mevalonate (MVA) pathway (Dai et al., 2012). Sesquiterpenes have been the prime targets in the field of synthetic biology, and several approaches have been used to enhance microbial production of sesquiterpenes. Overexpression of key genes in the MVA pathway (*tHMGR*, *UPC2.1*, and *ERG20*) to increase FPP flux, and replacement of promoter to downregulate *ERG9* to reduce this competing flux (Ro et al., 2006; Paddon et al., 2013), and codon optimization to alleviate translation inefficiency (Tokuoka et al., 2008) are the most commonly used ones. In addition, multicopy integration targeting repetitive chromosomal DNA sequences, and long terminal repeats of Ty element (*δDNA* sequence) to stabilize gene expression and to achieve high average copies (Lee and Da Silva, 1997; Tokuhiro et al., 2009; Tyo et al., 2009) were also employed.

**FIGURE 1:**
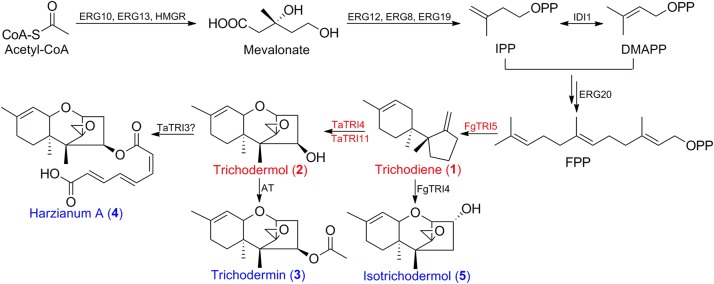
Biosyntheses of trichodermol and related sesquiterpenes by the endogenous MVA pathway in *S. cerevisiae.* Three enzymes are involved in trichodermol biosynthesis from FPP, trichodiene synthase FgTRI5 from *F. graminearum*, and cytochrome P450 monooxygenases, TaTRI4 and TaTRI11, from *T. arundinaceum.* Double arrows represented two steps catalyzed by the same enzyme. Red color annotated genes used in heterologous biosynthesis of trichodiene and trichodermol, red color annotated compounds synthesized in this work, and blue color annotated sesquiterpenes that could be prepared from trichodermol via post-modifications. AT, acetyltransferase.

Trichothecene sesquiterpenoids are produced by the fungal genera of *Fusarium*, *Stachybotrys*, *Myrothecium*, *Trichoderma*, and *Trichothecium* (McCormick et al., 2011), and are known to inhibit protein synthesis, and to induce oxidative stress, DNA damage, and cell cycle arrest in eukaryotic cells (Arunachalam and Doohan, 2013). Trichodermin (**3**; **Figure 1**) is a representative trichothecene produced by *Trichoderma brevicompactum* (Tijerino et al., 2011). It has attracted much attention due to significant inhibitory effects on some phytopathogenic fungi (Shentu et al., 2014), and potent but selective cytotoxicity toward several human tumor and normal cells (Su et al., 2013; Chien et al., 2017), suggesting that it is a promising candidate for the development of agrochemicals and antitumor agents. Although chemical modification of trichodermin has afforded new antifungal derivatives (Xu et al., 2013; Cheng et al., 2015), the lack of enough material and reactive sites in its structure has limited further modification and evaluations. Therefore, it is urgent to develop an alternative route for efficient generation of trichodermin and congeners for structure-activity relationship (SAR) study and further development. Since trichodermol (**2**; **Figure 1**) is the direct precursor for trichodermin, achievement of its heterologous biosynthesis is the first step in this endeavor.

The biosynthesis of trichodermol in *Trichoderma* spp. has been well-documented (Cardoza et al., 2011), proceeding with the same first step as other trichothecenes in *Fusarium* spp. (Paddon et al., 2011). The terpene cyclase TRI5 first catalyzes the conversion of FPP to the common precursor trichodiene (**1**; **Figure 1**), and then the enzymes involved in formation of the key intermediates trichodermol and isotrichodermol (**2** and **5**; **Figure 1**) diversify. In *F. graminearum*, a multifunctional cytochrome P450 monooxygenase FgTRI4 catalyzes four consecutive oxygenation steps to generate a series of intermediates, such as isotrichodermol or 12,13-epoxytrichothec-9-ene (McCormick et al., 2006; Tokai et al., 2007). While in *Trichoderma* spp., both TRI4 and TRI11 are required to oxidize trichodiene to trichodermol (Cardoza et al., 2015), and *TaTRI4* and *TaTRI11* have been individually expressed in *S. cerevisiae* as verified through feeding experiments (Cardoza et al., 2011). Subsequently, trichodermol was catalyzed by acetyltransferase and esterase to form trichodermin and harzianum A, respectively (**3** and **4**, **Figure 1**). Heterologous biosynthesis of trichodiene has been explored in *E. coli* (Hohn and Plattner, 1989) and transgenic tobacco (Hohn and Ohirogge, 1991), leading to trichodiene production of 60 μg L^-1^ and 5–10 ng gFW^-1^, respectively. Co-expression of *FgTRI5* and *FgTRI4* in yeast produced only the early intermediates in isotrichodermol biosynthesis (Tokai et al., 2007), while overexpression of *TaTRI5* and *TaTRI4* in *T. harzianum* resulted in the production of only precursor 12,13-epoxytrichothec-9-ene (Cardoza et al., 2015). Considering that heterologous biosynthesis of trichodermol remained unaccomplished, we primarily reconstructed and optimized its biosynthetic pathway in *S. cerevisiae* BY4741 to achieve its biosynthesis, and investigated the effect of produced trichodermol on the host by *de novo* RNA-Seq and qPCR analyses.

## Materials and Methods

### Strains and Culture Conditions

The strains used in this study were listed in **Table 1**. *F. graminearum* with complete trichothecene biosynthetic pathway was acquired from China General Microbiological Culture Collection (CGMCC) and grown on potato dextrose agar (PDA). *E. coli* DH5α for transformation and plasmid DNA extraction, and *E. coli* BL21 (DE3) for protein expression were cultured at 37°C in Luria-Bertani medium (10 g L^-1^ tryptone, 5 g L^-1^ yeast extract, and 10 g L^-1^ NaCl) supplemented with ampicillin (100 μg mL^-1^) or kanamycin (50 μg mL^-1^) if needed. *S. cerevisiae* BY4741 was provided by Prof. Yu Fu at Institute of Microbiology, and cultured at 30°C in yeast extract peptone dextrose (YPD; 10 g L^-1^ yeast extract, 20 g L^-1^ Bacto peptone, and 20 g L^-1^ glucose) or synthetic dextrose (SD; 20 g L^-1^ glucose and 0.67% yeast nitrogen base with ammonium sulfate, supplemented with appropriate nutrients) (Adams et al., 2000). D-(+)-Galactose (2%; w v^-1^) was used as an inducer when needed. Colonies for shake flask cultures were cultured in 5 mL SD medium at 30°C for 12 h, refreshed in 20 mL SD medium, grown until reaching mid-log phase, and the seed culture was inoculated into 100 mL SD medium with an initial OD_600_ value of 0.05 and cultured at 30°C, 220 rpm for 48 h.

**Table 1:** Strains and plasmids.

Strains and plasmids	Description	Source or reference
**Plasmids**		
pUC57-FgTRI5	Codon-optimized and synthesized *FgTRI5* from *F. graminearum* cloned into pUC57	Genscript
pUC57-TaTRI4	Codon-optimized and synthesized *TaTRI4* from *T. arundinaceum* cloned into pUC57	Genscript
pUC57-TaTRI11	Codon-optimized and synthesized *TaTRI11* from *T. arundinaceum* cloned into pUC57	Genscript
pET30a	Protein expression plasmid in *E. coli*	Novagen
pET30a-FgTRI5	*FgTRI5* cloned and inserted into the *Not* I site of pET30a	This study
pESC-URA	Episomal expression plasmid in *S. cerevisiae*	Dejong et al., 2006
pESC-FgTRI5	pESC-URA derivative with *FgTRI5*	This study
pESC-TaTRI4-TaTRI11	pESC-URA derivative with codon-optimized *TaTRI4* and *TaTRI11*	This study
pLLeu-tHMGR-UPC2.1	Plasmid with *tHMGR* and *UPC2.1*	Dai et al., 2012
pRS303::VC	Parent plasmid for pRS303ap	Addgene
pRS303ap	Integration plasmid including *δDNA* locus of *S. cerevisiae*	This study
pRS303ap-FgTRI5	pRS303ap derivative with *FgTRI5*	This study
pRS303ap-FgTRI5-O	pRS303ap derivative with codon-optimized *FgTRI5*	This study
**Strains**		
*E. coli* BL21 (DE3)	F^-^ *ompT hsdS*_B_ (r^-^_B_m^-^_B_) *gal dcm*	CWBIO
*S. cerevisiae* BY4741	*MAT*a *his3*Δ1 *leu2*Δ0 *met15*Δ0 *ura3*Δ0	Brachmann et al., 1998
TD0	BY4741 transformed with plasmid pESC-URA	This study
TD1	BY4741 transformed with plasmid pESC-FgTRI5	This study
TD2	BY4741 transformed with plasmids pESC-FgTRI5 and pLLeu-tHMGR-UPC2.1	This study
TD3	BY4741 integrated with *FgTRI5* into *δDNA* locus	This study
TD4	BY4741 integrated with codon-optimized *FgTRI5* into *δDNA* locus	This study
TD5	TD4 integrated with codon-optimized *TaTRI4* and *TaTRI11* into the *rDNA* locus	This study


### Plasmids

Plasmids and primers used in this study were listed in **Table 1** and **Supplementary Table 1**. DNA sequences of *FgTRI5* (from *F. graminearum*), *TaTRI4* (GenBank Accession No. FN394495.1), and *TaTRI11* (GenBank Accession No. FN394493.1), were codon-optimized according to the codon bias of yeast (**Supplementary Table 2**), synthesized by Genscript (Nanjing, China), and delivered as a series of pUC57 plasmids (**Table 1**). Episomal plasmid pESC-URA was obtained from Hangzhou Biosci Biotech Co. (Hangzhou, China). Plasmids pLLeu-tHMGR-UPC2.1 and pBlue-FLAG-URA3-FLAG were kind gifts from Profs. Xueli Zhang at Tianjin Institute of Industrial Biotechnology, and Huiqiang Lou at China Agricultural University, respectively.

### Strain Construction

Oligonucleotides used to amplify and clone the cDNA of *FgTR15* were given in **Supplementary Table 1**. cDNA of *FgTRI5* was cloned and inserted into the *Not* I site of pET30a to generate an expression plasmid pET30a::FgTRI5, from which *FgTRI5* was cloned and inserted into the *BamH* I/*Kpn* I sites of pESC-URA under the control of promoter *GAL1* and terminator *CYC1* to construct pESC::FgTRI5. Plasmid pRS303ap, derived from pRS303::VC, was prepared following the procedures below (**Supplementary Figure 3**). The left and right DNA sequences of *HIS3* auxotrophic marker were amplified by PCR and assembled by overlap extension PCR (OE-PCR) to generate pRS303SL, into which the *δDNA* locus with added restriction enzyme site was inserted to prepare pRS303SL-δDNA. The divided *δDNA* loci, *δDNA*1 and *δDNA*2, were separately amplified from genomic DNA of BY4741 and assembled by OE-PCR to generate new *δDNA*. Multiple clone site (MCS) *Xho* I-*Not* I-*Sac* II-*BamH* I was added between *δDNA*1 and *δDNA*2, and *Hpa* I was added to the 5′ and 3′ ends. Newly prepared *δDNA* was inserted into the *Kpn* I/*Sac* I site of pRS303SL to generate pRS303SL-*δ*DNA, of which a new selection marker 5FLAG-URA3-5FLAG cloned from pBlue-FLAG-URA3-FLAG was inserted into the *Xho* I site to prepare pRS303a. Bidirectional promoter *GAL1*/*GAL10p* and terminator *CYC1t* were amplified from pESC-URA and assembled by OE-PCR to generate fragment GAL1/GAL10p-CYC1t with *SexA* I added in between, which was inserted into the *Not* I site of pRS303a to afford pRS303ap. Insertion of *FgTRI5* and the codon-optimized *FgTRI5* into the *SexA* I site of pRS303ap generated pRS303ap-FgTRI5 and pRS303ap-FgTRI5-O, respectively.

To construct TD5, the codon-optimized *TaTRI4* and *TaTRI11* were separately amplified from pUC57-TaTRI4 and pUC57-TaTRI11, and inserted into the *BamH* I/*Sal* I and *EcoR* I sites of pESC-URA to generate pESC-TaTRI4-TaTRI11, from which fragment CYC1t-TaTRI4-GAL1p-GAL10p-TaTRI11-ADH1t was amplified. Chromosomal DNA of BY4741 was used as the template for PCR amplification of *rDNA*1 and *rDNA*2, and pRS303::VC as the template to amplify the *HIS3* marker. Purified PCR products of CYC1t-TaTRI4-GAL1p-GAL10p-TaTRI11-ADH1t and *rDNA*1 were used as the templates for secondary PCR in generation of rDNA1-TaTRI4-TaTRI11, and those of *HIS3* and *rDNA*2 as the templates to generate HIS3-rDNA2. Co-transformation of rDNA1-TaTRI4-TaTRI11 and HIS3-rDNA2 into TD4 generated rDNA1-TaTRI4-TaTRI11-HIS3-rDNA2, which was inserted into the *rDNA* locus of TD4 via homologous recombination to afford TD5. Constructed plasmids and fragments were verified by DNA sequencing, and transformed into yeast accordingly, using standard electroporation method (Becker and Guarente, 1991).

### *In vitro* Enzymatic Assay of FgTRI5

*E. coli* BL21 (DE3) harboring pET30a::FgTRI5 was grown at 37°C in LB medium until the OD_600_ value reached 0.4–0.6, IPTG (Sigma-Aldrich, St. Louis, MO, United States) was added to a final concentration of 0.3 mM, and further incubated at 16°C, 170 rpm for 12 h. Cells were harvested by centrifugation and sonicated on ice. The His_6_-tagged FgTRI5 was purified by Ni-NTA agarose chromatography and protein purity was assessed by Coomassie blue staining after SDS-PAGE (sodium dodecyl sulfate polyacrylamide gel electrophoresis) on a 10% polyacrylamide gel (Pan et al., 2011). Purified protein was concentrated with Amicon Ultra-0.5 mL Centrifugal Filters (Millipore, Billerica, MA, United States), with its concentration determined using the BCA Protein Assay Kit (Vazyme, Nanjing, China), and stored in 5% glycerol at -80°C.

Enzymatic assay of FgTRI5 was performed according to published procedures (Vedula et al., 2007, 2008). FPP (500 μM; Sigma-Aldrich, St. Louis, MO, United States) was incubated with 350 μg purified FgTRI5 in 4 mL buffer (10 mM Tris, 5 mM MgCl_2_, 15% glycerol, and 5 mM β-mercaptoethanol; pH 7.8), using a solution without FgTRI5 as the negative control. Buffer solution was overlaid with *n*-pentanes (Sinopharm Chemical Reagent Co. Ltd., Shanghai, China) in a glass tube at 30°C for 24 h, the reaction products were extracted with *n*-pentanes, and purified on a 200–300 mesh silica gel column. After concentration, purified extracts were analyzed by gas chromatography-mass spectrometry (GC/MS).

### Western Blot

Protein extracts from TD1 and TD5 cells were dissolved in PBS buffer (137 mM NaCl, 2.7 mM KCl, 10 mM Na_2_HPO_2_, and 2 mM KH_2_PO_4_), loaded to 10% SDS-PAGE, and the separated proteins were electro-transferred onto polyvinyl difluoride (PVDF) membranes (Millipore, Billerica, MA, United States) and probed with appropriate antibodies. For detection of His_6_-tagged FgTRI5, the HRP-labeled 6 × His monoclonal antibody was used for immunoblotting. To detect proteins TaTRI4 (fused with c-myc-tag) and TaTRI11 (fused with FLAG-tag), membranes were separately incubated with the primary (c-myc-tag and FLAG-tag monoclonal antibodies) and secondary (peroxidase-labeled antibody to mouse IgG) antibodies (Proteintech, Rosemont, IL, United States) in order, followed by detection with enhanced chemiluminescence (Thermo Fisher Scientific, Waltham, MA, United States).

### Preparation of Authentic Trichodermol

Authentic trichodermol was prepared by hydrolysis of trichodermate A according to a published procedure (Li et al., 2016). Sodium methoxide (MeONa; 1.1 mg) was added to a 2 mL solution of 9:1 dichloromethane (CH_2_Cl_2_)-methanol (MeOH) containing 2 mg trichodermate A, the solution was stirred at room temperature (RT) for 3 h, and the solvents were removed under vacuum. The residue was extracted with CH_2_Cl_2_ for three times, and the organic solvent was evaporated to dryness under vacuum. The product was purified by reversed phase HPLC (Agilent Technologies, Santa Clara, CA, United States) equipped with an Agilent Zorbax SB-C18 column (9.4 mm × 250 mm; 5 μm; 45-75% acetonitrile (CH_3_CN) in H_2_O for 30 min; 2 mL min^-1^), and characterized based on HRESIMS (m/z 251.1642; *calcd.* for C_15_H_22_O_3_) and ^1^H-NMR data (**Supplementary Figure 4**) (Li et al., 2016).

### Identification and Quantification of Trichodiene

After incubation for 48 h, whole cell cultures of strains TD1-TD4 were extracted with analytical CH_2_Cl_2_ for three times, the organic solvents were evaporated to dryness under vacuum, and the residues were dissolved in CH_2_Cl_2_ for GC and GC-MS analyses (Dickschat et al., 2011). Trichodiene was identified using an Agilent 6890N GC coupled with an Agilent 5975 inert XL mass-selective detector (MSD) with a HP-5MS column (25 m × 0.20 mm; 0.33 μm). The oven temperature was set at 60°C for 2 min, increased by 10°C min^-1^ to 290°C, and held at 290°C for 4 min. The injector temperature was set at 260°C. Helium was used as the carrier gas (0.8 mL min^-1^) in the splitless mode. Trichodiene was characterized by comparison of its MS data with those published (Dickschat et al., 2011), and quantified by integrating its peak area in GC chromatogram and comparing to an internal standard, (±)-mevalonolactone (Sigma-Aldrich, St. Louis, MO, United States), which showed similar volatility, but different retention time to trichodiene or other volatiles from yeast.

### Identification of Trichodermol

After incubation for 48 h, whole cell culture, supernatant, and TD5 cells were separately extracted with equal volume of ethyl acetate (EtOAc), and the extracts were analyzed by an Agilent Accurate-Mass-Q-TOF LC/MS 6550 instrument equipped with an electrospray ionization (ESI) source. HPLC separation was performed on an Agilent Eclipse Plus C-18 RRHD column (2.1 × 50 mm; 1.8 μm) using 0.1% formic acid in H_2_O (A) and CH_3_CN (B) as the eluents (20% B for 0.8 min, 20–100% B for 14.2 min, and 100% B for 3 min; 0.3 mL min^-1^). For MS analysis, the fragmentor and capillary voltages were 175 and 3,500 V, respectively. Nitrogen was supplied as the nebulizing and drying gas, and the temperature and flow rate of the drying gas were 200°C and 14 L min^-1^, respectively. The sheath gas temperature and flow were set at 350°C and 11 L min^-1^, respectively. The pressure of the nebulizer was 35 psi. The instrument was tuned for a range of 40–1,700 m/z at 1 spectra s^-1^. All MS experiments were performed in positive ion mode. Trichodermol was verified by comparison of the extracted ion spectrum and MS fragments with those of an authentic sample using MassHunter Qualitative Analysis B.07.00, and the yield was estimated by comparison of the MS peak area to the standard curve prepared using an authentic sample (Li and Smolke, 2016).

### RNA Isolation and qPCR Analysis

After induction with 2% D-(+)-galactose for up to 48 h, the total RNAs were isolated from TD5 and BY4741. Yeast cells were collected, frozen quickly in liquid nitrogen, and the RNAs were extracted with Yeast RNA Kit (Omega Bio-tek Inc., San Francisco, CA, United States) according to the manufacturer’s protocol. The quality and quantity of RNAs were examined using Nanodrop 2000 spectrophotometer (Thermo Scientific, Waltham, MA, United States), and verified by agarose gel electrophoresis. RNA samples were treated with RQ1 RNase-Free DNase (Promega, Madison, WI, United States) to remove chromosomal DNA. After PCR verification, a 0.4 μg sample was reversely transcribed using PrimeScript RT Master Mix (Takara Biotechnology, Dalian, China), and subjected to qPCR analysis using a Light Cycler 96 qPCR instrument (Roche, Basle, Switzerland). Each reaction (20 μL) contained 1 μL reversely transcribed DNA, 0.4 μM forward and reversed primers, and 10 μL SYBR Green PCR Master Mix (CWBIO, Beijing, China). Reactions were maintained at 95°C for 600 s, followed by 40 cycles of three step amplifications at 95°C for 10 s, 55°C for 10 s, and 72°C for 32 s, and then melted at 95°C for 10 s, 65°C for 60 s, and 97°C for 1 s. Fluorescence was measured at the end of each cycle with *ACT1* as an internal control, and analyzed using the 2^-ΔΔCT^ method (Zha et al., 2014). All data were calculated from three independent experiments, and presented as mean ± SD. Statistical analyses were performed using analysis of variance (ANOVA) with GrapdhPad_Prism_6.0 software, and *P*-values < 0.05 were considered as statistically significant (^∗^*P* < 0.05, ^∗∗^*P* < 0.01, ^∗∗∗^*P* < 0.001).

### Measurement of Integration Efficiency and Quantification of Integrated Gene Copies

The genomic DNAs of TD3–TD5 were extracted and quantified using Nanodrop 2000 spectrophotometer. Integration efficiency was measured by comparison of Integrated Density Value (IDV) between the target genes and actin *ACT1* (Urnov et al., 2005). PCR analyses were performed using the oligonucleotides designed between the target genes and integration loci (**Supplementary Table 1**).

qPCR was employed to quantify the copies of integrated genes (Cardoza et al., 2015). A standard curve assay was performed using concentrations of 1, 2, 4, 8, 16, and 32 ng μL^-1^ for each genomic DNA, and the oligonucleotides designed for actin *ACT1* and the integrated genes in different strains (**Supplementary Table 1**). The amounts of target genes and *ACT1* (ng μL^-1^) for each genomic DNA were calculated based on the equations derived from the calibration curves. By calculating the ratio of the target genes to *ACT1*, copies of the target genes could be determined.

## Results

### Biosynthesis of Trichodiene in *S. cerevisiae*

**FIGURE 2:**
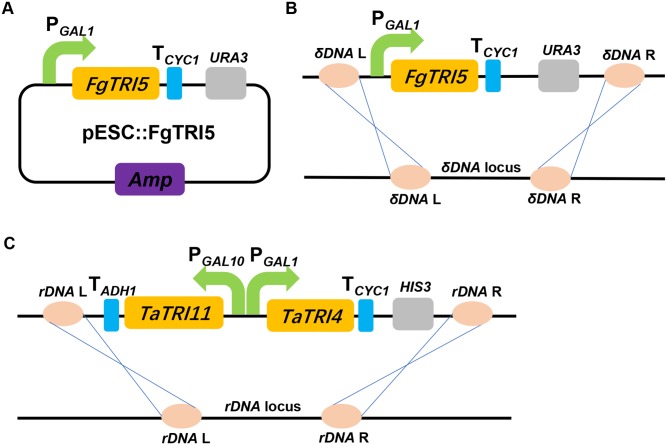
A schematic representation of the strategies used to construct the expression cassettes for *FgTRI5*, *TaTRI4*, and *TaTRI11*. **(A)** Construction of *FgTRI5* expression cassette using an episomal plasmid pESC-URA. **(B)** Integration of *FgTRI5* expression cassette into the *δDNA* locus of the chromosome. **(C)** Integration of *TaTRI4* and *TaTRI11* expression cassettes into the *rDNA* locus of the chromosome.

*S. cerevisiae* BY4741 (**Table 1**), a derivative of strain S288C (Brachmann et al., 1998) and the host for miltiradiene and strictosidine biosyntheses (Zhou et al., 2012; Brown et al., 2015), was selected for heterologous biosynthesis trichodiene. Evaluation of *in vitro* enzymatic activity of the His_6_-tagged FgTRI5 synthase from *E. coli* BL21 (DE3) verified that the it catalyzed the conversion from FPP to trichodiene (Methods; **Supplementary Figure 1**). A recombinant gene encoding His_6_-tagged FgTRI5 synthase was introduced into strain BY4741 using the episomal plasmid pESC::FgTRI5 (**Table 1** and **Figure 2A**). The resulting transformants were selected using SD-URA plate, verified by PCR, and the correct one was named strain TD1. Although Western blot analysis revealed expression of *FgTRI5* in TD1 (**Supplementary Figure 2**), neither trichodiene nor any other intermediates was detected by GC-MS after incubation for 48 or 96 h (data not shown), possibly due to insufficient supply of FPP.

**FIGURE 3:**
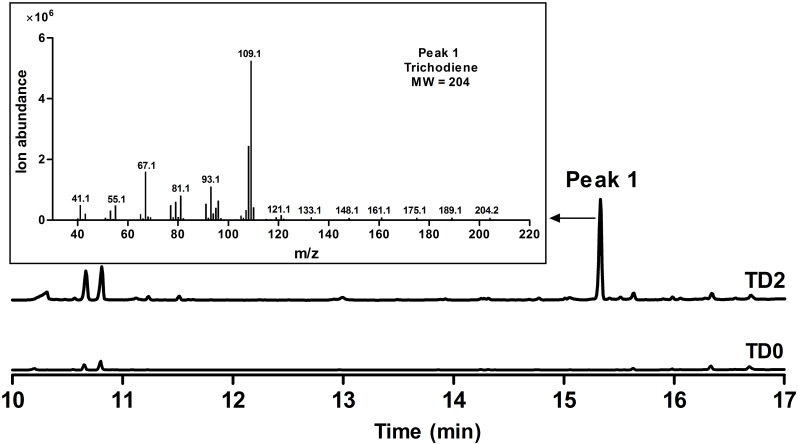
GC and GC-MS analyses of whole cell culture extracts of TD0 and TD2. Total ion chromatograms (TICs) of whole cell culture extract of TD2 (top) and the control TD0 (bottom). Peak 1 was a newly observed product in TD2, with its mass spectrum shown. TD0 harbored the empty plasmid pESC-URA, while TD2 harbored pESC::FgTRI5 and pLLeu-tHMGR-UPC2.1, which were cultured in SD-URA and SD-URA-LEU media, respectively, supplemented with 2% D-(+)-galactose for 48 h.

To increase FPP accumulation, the plasmid pLLeu-tHMGR-UPC2.1 (Dai et al., 2012) was transformed into TD1 to overexpress *tHMGR* and *UPC2.1*. The resulting strain TD2 was cultured in SD-URA-LEU medium for 48 h, and the whole cell culture was extracted with CH_2_Cl_2_ to obtain the organic phase for GC-MS analysis. In the GC chromatogram, a peak was observed at 15.3 min, showing the same molecular mass and fragments as those of trichodiene (Dickschat et al., 2011), which was absent in the CH_2_Cl_2_ phase of the cultured control strain TD0 with an empty plasmid (**Figure 3**). A titer of 683 μg L^-1^ was determined for trichodiene synthesized in TD2 based on the results from GC analysis of the organic phase.

### Production Improvement by Genomic Integration and Codon Optimization

**FIGURE 4:**
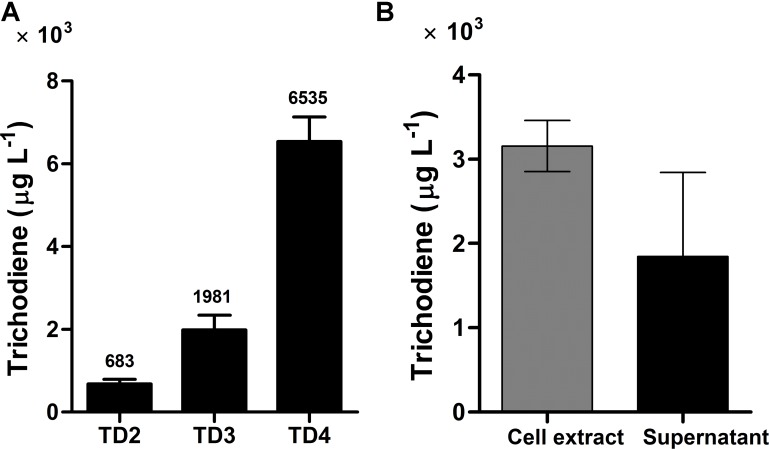
Trichodiene production in different strains and its distribution in yeast cell extract and supernatant. **(A)** Trichodiene production in TD2–TD4. Numbers above the bars represented the yield of trichodiene (μg L^-1^). **(B)** Distribution of produced trichodiene in yeast cell extract and supernatant. Error bars were calculated from three independent experiments. TD2 harbored the plasmids pESC::FgTRI5 and pLLeu-tHMGR-UPC2.1. TD3 and TD4 were integrated with *FgTRI5* and the codon-optimized *FgTRI5*, respectively. TD2 was cultured in SD-URA-LEU medium, while TD3 and TD4 were cultured in SD-URA medium, all supplemented with 2% D-(+)-galactose for 48 h.

Expression of *FgTRI5* using an episomal plasmid with increased FPP supply led to the production of trichodiene, but the yield was rather low to proceed further in trichodermol biosynthesis. Since *de novo* chromosomal engineering is generally considered as a more robust approach for expression of genetic constructs compared to those using the artificial plasmid-based systems (Tokuhiro et al., 2009; Tyo et al., 2009), an integration plasmid pRS303ap was constructed to express *FgTRI5* (**Supplementary Figure 3**). With a *URA3* selection marker flanked by two repeated FLAG sequences to recycle *URA3* in 5-fluroorotic acid (5-FOA) plate (Ro et al., 2006), the plasmid can repeatedly integrate multiple genes into the dispersed chromosomal *δDNA* locus of yeast (Lee and Da Silva, 1997). After linearization with *Hpa* I and transformation of pRS303ap::FgTRI5 into the host (**Figure 2B**), the resulting transformant TD3 was selected and cultured in SD-URA medium. The level of trichodiene in the CH_2_Cl_2_ phase of the whole cell culture was detected to be 1,981 μg L^-1^ by GC after 48 h incubation, which was a 1.9-fold increase compared to that produced by TD2 (**Figure 4A**).

Codon optimization is a typical approach to enhance expression of heterologous genes containing rarely used codons in host organisms (Tokuoka et al., 2008). To test whether it plays such a role in trichodiene biosynthesis, the DNA sequence of *FgTRI5* was optimized and synthesized according to the codon bias of yeast (Methods; **Supplementary Table 2**). The optimized *FgTRI5* was cloned downstream of the galactose-regulated promoter *GAL1* in pRS303ap to generate pRS303ap::FgTRI5-O, from which the target PCR fragment was amplified and transformed into BY4741. The resulting transformant TD4 was selected and incubated in SD-URA medium. Compared to trichodiene production in TD2, an increase of 8.6-fold was achieved in TD4 (**Figure 4A**), reaching a maximum of 6,535 μg L^-1^ at 48 h, indicating that codon optimization indeed enhanced the expression of *FgTRI5* in BY4741. In addition, 36.9% of produced trichodiene was detected in the supernatant at 48 h (**Figure 4B**).

To increase FPP supply in TD4, *tHMGR* and *UPC2.1* were overexpressed by introducing pLLeu-tHMGR-UPC2.1 or integrating the two genes into the *δDNA* locus of BY4741, and by replacing the promoter of *ERG9* with a copper-regulated, *ERG9*-repressing *P_CTR3_*. Although significant increase in *tHMGR* and *UPC2.1* transcription and apparent decrease in *ERG9* expression were detected by qPCR, GC-MS analysis of the incubation products revealed that these approaches failed to further improve the production of trichodiene (data not shown).

### Construction of Trichodermol Biosynthetic Pathway in *S. cerevisiae*

**FIGURE 5:**
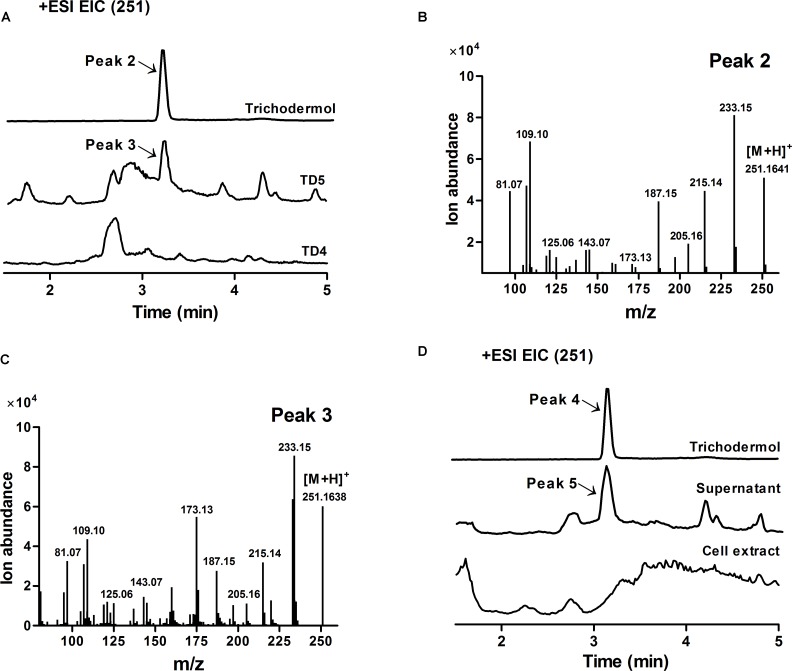
Verification of trichodermol production in TD5 by HPLC-MS. **(A)** Extracted ion chromatograms (EICs) of authentic trichodermol, whole cell culture extract of TD5, and extract of the control TD4. Peaks 2 and 3 represented authentic trichodermol and the newly observed product in TD5, respectively. **(B)** Mass spectrum of authentic trichodermol (peak 2). **(C)** Mass spectrum of newly observed product in TD5 (peak 3). **(D)** EICs of authentic trichodermol, the supernatant of TD5, and the cell extract of TD5. Peak 4 represented authentic trichodermol, showing identical mass spectrum to peak 2. Peak 5 represented the newly observed product in the supernatant of TD5, showing identical mass spectrum to peak 3. TD5 with the codon-optimized *FgTRI5*, *TaTRI4*, and *TaTRI11* was cultured in SD-URA-HIS medium, while TD4 with the codon-optimized *FgTRI5* was cultured in SD-URA medium, both supplemented with 2% D-(+)-galactose for 48 h.

Trichodermol, the key precursor in trichodermin and harzianum A biosyntheses, was generated from trichodiene via consecutive reactions catalyzed by cytochrome P450 monooxygenases TRI4 and TRI11 (Cardoza et al., 2015). Since TD4 produced the highest level of trichodiene, the biosynthetic pathway was constructed in this strain. After codon optimization (Methods; **Supplementary Table 2**), *TaTRI4* (with c-myc-tag) and *TaTRI11* (with FLAG-tag) were integrated into the *rDNA* locus of TD4 by homologous recombination (Dahm and Jennewein, 2010; Dai et al., 2013; Yan et al., 2014), and expressed under the control of the galactose-regulated promoters, *GAL1* and *GAL10*, respectively (**Figure 2C**). The resulting strain TD5 was cultured in SD-URA-HIS medium at 30°C for 48 h using TD4 as the control, and Western blot results showed that both *TaTRI4* and *TaTRI11* expressed in TD5 (**Supplementary Figure 2**). The EtOAc extracts of whole cell cultures of TD4 and TD5 were analyzed by high performance liquid chromatography-mass spectrometry (HPLC-MS), and trichodermol production was verified by comparison of the extracted ion spectrum of the target product with an authentic sample. Specifically, a peak with the same molecular mass and MS fragments as authentic trichodermol was found in the extracted ion chromatogram (EIC) of the whole cell culture extract of TD5 (**Figures 5A–C**), indicating that trichodermol is synthesized via the biosynthetic pathway reconstructed in TD5, with an estimated titer of 252 μg L^-1^ at shake flask level. In addition, analysis of the EICs for both culture supernatant and cell extract of TD5 revealed that trichodermol was exported entirely out of the cells (**Figure 5D**).

### Measurement of Integration Efficiency and Quantification of Integrated Gene Copies

Since recombination frequency depends on integration efficiency of the integrated genes and their physical distance, it may vary considerably for different genes (Lacks, 1966). The efficiencies to integrate the heterologous genes into the target loci were measured, and those of 31.7, 17.4, 20.1, and 10.0% were calculated for *FgTRI5*, the optimized *FgTRI5*, *TaTRI4*, and *TaTRI11*, respectively (**Supplementary Figure 5**).

qPCR is a rapid, sensitive, and accurate technique to quantify the copies of transgenes (Batista et al., 2014). qPCR results showed that one or two copies of *FgTRI5*, two or three copies of optimized *FgTRI5*, six or seven copies of *TaTRI4*, and one or two copies of *TaTRI11* were integrated into respective strains (**Supplementary Figure 6**).

### RNA-Seq and qPCR Analyses of Trichodermol Biosynthesis-Related Genes in TD5

RNA-Seq is a recently developed high-throughput technology to simultaneously quantify expression of thousands of genes in comprehensive transcriptome studies (Wang et al., 2009; Khatoon et al., 2014; Ye et al., 2017). Here, the complete transcriptome of TD5 was analyzed by RNA-Seq in comparison with BY4741, and the raw data were deposited (NCBI accession number: SRP148433). The differential expression genes (DEGs) were screened (**Supplementary Data Sheet 1**) according to the FPKM (fragments per kilobase of exon model per million mapped reads) value, and annotated through GO (Gene Ontology Consortium) and KEGG (Kyoto Encyclopedia of Genes and Genomes) function classifications (**Supplementary Figures 7–9**). The annotated DEGs were classified into categories of biological processes, cellular components, and molecular functions, with high percentage of unigenes involved in the functions of metabolic progress, and binding and catalytic activities (**Supplementary Figure 8**). To explore their biological functions, the DEGs were assigned to the metabolic pathways described in KEGG database, including metabolism, environmental information processing, cellular processes, and genetic information processing (**Supplementary Figure 9**). The unigenes belong to terpene metabolic and membrane transport pathway attracted our attention since they are likely involved in the biosynthesis and export of trichodermol. Therefore, those involved in ergosterol biosynthetic pathway and ATP-binding cassette (ABC) transporters were selected and verified by qPCR (see **Supplementary Table 1** for the primers used).

**FIGURE 6:**
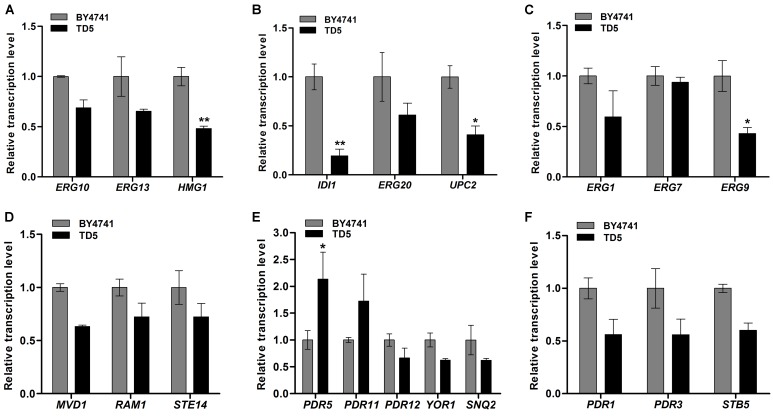
Relative transcription levels of the differential expression genes (DEGs) in TD5 in comparison with BY4741 revealed by qPCR analysis. Relative transcription levels of the genes involved in MVA biosynthesis **(A)**, FPP biosynthesis **(B)**, ergosterol biosynthesis **(C)**, terpenoids metabolism **(D)**, ABC-transporters **(E)**, and ABC-transporter transcription factors **(F)**. TD5 was constructed by integrating heterologous *FgTRI5*, *TaTRI4*, and *TaTRI11*, and cultured in SD-URA-HIS medium, while BY4741 was cultured in SD medium, both supplemented with 2% D-(+)-galactose for 48 h. The data were calculated from three independent experiments and presented as mean ± SD (^∗^*P* < 0.05, ^∗∗^*P* < 0.01).

Ergosterol biosynthetic pathway includes three modules responsible for the biosynthesis of MVA, FPP, and ergosterol, respectively (**Supplementary Figure 10**) (Caspeta et al., 2014; Hu et al., 2017). The first module starts with the condensation of acetyl-CoA catalyzed by ERG10, ERG13, and HMG-CoA (HMG1 and HMG2) reductases to produce MVA (Hu et al., 2017), and qPCR analysis showed that their encoding genes were downregulated in TD5 compared to those in BY4741, in which *HMG1* was significantly downregulated (*P* < 0.01; **Figure 6A** and **Supplementary Data Sheet 2**). The second one is responsible for FPP biosynthesis from MVA, involving six successive reactions individually catalyzed by ERG12, ERG8, ERG19, IDI, and ERG20 (Hu et al., 2017), and the representative genes *IDI* and *ERG20*, especially *IDI* (*P* < 0.01; **Supplementary Data Sheet 2**), were downregulated in TD5 (**Figure 6B**). Compared to the first two modules, the third one is more complex, and *ERG9*, *ERG1*, *ERG7*, and *ERG11* are involved in the early steps of ergosterol biosynthesis and considered as the essential genes (Hu et al., 2017). Analysis of the first three genes in this module revealed that *ERG9* and *ERG1* were downregulated, and *ERG9* were significantly downregulated (*P* < 0.05; **Supplementary Data Sheet 2**), while *ERG7* was nearly unaffected (**Figure 6C**). On the other hand, the terpene metabolism-related genes, *RAM1*, *STE14*, and *MVD1*, were all downregulated in TD5 (**Supplementary Table 3** and **Figure 6D**).

ABC transporters have attracted attention since they can transport toxic compounds out of cells or into vacuoles/lysosomes (Tsujimoto et al., 2013). Those involved in pleiotropic drug resistance (PDR) have been studied in yeast, which is known to express various ABC transporters (Matsufuji et al., 2010; Shahi et al., 2010; Tsujimoto et al., 2013, 2015; Gupta et al., 2014; Demir and Koc, 2015). The ABC transporters in TD5 were screened to identify those involved in transporting synthesized trichodermol. Only the encoding gene *PDR5* (*P* < 0.05; **Supplementary Data Sheet 2**) was significantly upregulated, while the remaining ones were all downregulated (**Supplementary Table 3** and **Figures 6E,F**), suggesting that PDR5 might play a role in transporting trichodermol to culture supernatant independent of any other heterologous efflux pumps.

## Discussion

Heterologous biosynthesis of natural products as therapeutics or drug leads has attracted much attention due to the success in microbial production of artemisinic acid using engineered yeast (Ro et al., 2006). Recently, opioids and anticancer alkaloid noscapine were also synthesized in yeast (Galanie et al., 2015; Li and Smolke, 2016). Although heterologous biosynthesis of trichodiene, the precursor for trichothecene sesquiterpenes, have been explored in *E. coli* (Hohn and Plattner, 1989) and transgenic tobacco (Hohn and Ohirogge, 1991), the yields were relatively low. Co-expression of *FgTRI5* and *FgTRI4* in yeast produced 2a-hydroxytrichodiene, 12,13-epoxytrichothec-9-ene, 12,13-epoxy-9,10-trichoene-2a-ol, isotrichodiol, and isotrichotriol, which are the early intermediates in isotrichodermol biosynthesis (Tokai et al., 2007). While overexpression of *TaTRI5* and *TaTRI4* in *T. harzianum* led to the production of only 12,13-epoxytrichothec-9-ene (Cardoza et al., 2015).

*S. cerevisiae* BY4741 was selected as the host for trichodermol biosynthesis due to its inherent advantages, such as availability of correctly configured heme-containing P450s and reductases for expressions (Duan and Schuler, 2006; Paddon and Keasling, 2014). Using the endogenous MVA pathway, we reconstructed the biosynthetic pathway of trichodermol and achieved its first heterologous biosynthesis. We also demonstrated the effectiveness of codon optimization and integration expression for optimization and expression of heterologous genes (Tokuhiro et al., 2009; Tyo et al., 2009; Presnyak et al., 2015). Using an episomal plasmid with overexpressed *tHMGR* and *UPC2.1*, trichodiene was produced at a titer of 683 μg L^-1^. Considering that episomal plasmids may suffer from genetic instability (Tyo et al., 2009), a multicopy integration vector pRS303ap targeting the *δDNA* sequence was constructed, and a 1.9-fold increase in trichodiene production was achieved, suggesting that integration expression might stabilize gene duplication and result in higher expression of *FgTRI5* (Tokuhiro et al., 2009; Tyo et al., 2009). Since codon optimization has been reported to enhance translational efficiency in microbial production of sesquiterpenes (Tokuoka et al., 2008), a trichodiene synthase gene was synthesized according to the codon preference of yeast and inserted into the *δDNA* sequence of BY4741. Integration expression of the optimized *FgTRI5* in BY4741 led to an 8.6-fold increase in trichodiene production compared to that using an episomal plasmid. Although expression of the codon-optimized *TaTRI4* and *TaTRI11* in TD4 using an episomal plasmid failed to synthesize trichodermol (data not shown), integration of *TaTRI4* and *TaTRI11* into the repetitive chromosomal *rDNA* sequence of the same strain resulted in trichodermol production as verified by HPLC-MS analysis. Although the heterologous genes were effectively integrated into the target loci of genome, the integration efficiencies and gene copies varied for transgenes, possibly resulting from different integration loci and the effects of transgenes on host. In addition, it remained to be clarified whether the gene expression and trichodermol production correlate with the integration efficiency and gene copies.

Since the commonly used small peptide tags, FLAG-, poly-His-, and c-myc-tag have been reported to have minimal effects on the tertiary structure and biological activity (Bucher et al., 2002; Terpe, 2003; Zhao et al., 2013), the tagged enzymes were used in trichodermol biosynthesis for better detection in Western blot experiments.

Although the first heterologous biosynthesis of trichodermol was achieved in BY4741, the relationship between the MVA flux and the yields of trichodiene and trichodermol remained unclear. It has been reported that overexpression of *tHMGR*, *UPC2.1*, and *ERG20*, and downregulation *ERG9* favored terpenoid production (Ro et al., 2006; Dai et al., 2012; Zhou et al., 2012; Paddon et al., 2013; Brown et al., 2015), but we found that increase in FPP flux enhanced trichodiene production only at titers lower than 6,535 μg L^-1^, possibly due to the negative feedback regulation of sesquiterpene biosynthesis exerted by the produced trichodiene and trichodermol, as revealed by RNA-Seq and qPCR analyses. In addition, analysis of the culture supernatant and cell extract of TD4 and TD5 revealed that trichodermol was completely exported out of cells, compared to only 36.9% for trichodiene, leading to speculation that the produced trichodiene inhibited the growth of yeast cells, which implied the necessity to overexpress certain transporter genes (e.g., *TRI12* from *Fusarium* spp.) to facilitate transportation of the product out of the cells (Alexander et al., 1999). On the other hand, cytochrome P450 monooxygenases TaTRI4 and TaTRI11 are key enzymes involved in trichodermol biosynthesis, and further optimization is necessary to improve their expression and enzymatic activity. Since the heterologous pathway introduced into yeast including more P450s may lead to decreased conversion efficiency of each P450, cognate reductases once reported to increase the activity of P450s could be the target for exploration in further study (Paddon et al., 2013; Li and Smolke, 2016).

## Conclusion

In the current study, we first synthesized trichodiene, the common precursor of trichothecene sesquiterpenes in *S. cerevisiae* BY4741, and improved its production to 6,535 μg L^-1^ by heterologous expression of the codon-optimized *FgTRI5*. We further achieved the first heterologous biosynthesis of trichodermol, a key scaffold for the generation of diverse fungal sesquiterpenoids by reconstructing its biosynthetic pathway in BY4741. We also revealed that trichodermol downregulated the genes involved in ergosterol biosynthesis, but significantly upregulated *PDR5* related to membrane transport pathway in *S. cerevisiae* through RNA-Seq and qPCR analyses, which provided clues for further improvement of trichodermol production in future study. This work demonstrated the feasibility to produce this class of fungal natural products by heterologous biosynthesis in yeast. Through further optimization, the reconstructed pathway will serve as a platform for efficient generation of the trichodermin derivatives as potential candidates for agrochemicals and antitumor agents.

## Author Contributions

YC conceived the study. JL and YNZ performed the genetic and transcription experiments and analyzed the primary data. JL drafted the manuscript. YGZ and SZ performed the chemical experiments and structure characterizations. GL and YC supervised the whole work and revised the manuscript. All authors read and approved the final manuscript.

## Conflict of Interest Statement

The authors declare that the research was conducted in the absence of any commercial or financial relationships that could be construed as a potential conflict of interest.
