# Supplementary material for: Heterologous Biosynthesis of the Fungal Sesquiterpene Trichodermol in Saccharomyces cerevisiae
Source: Front Microbiol. 2018 Aug 6;9:1773. doi: 10.3389/fmicb.2018.01773 (PMC6087768; doi:10.3389/fmicb.2018.01773)
Supplement: Supplementary file 1 [file Presentation_1.pdf]

Supplementary Material

Heterologous Biosynthesis of the Fungal Sesquiterpene Trichodermol in *Saccharomyces cerevisiae*

Jianghua Liu, Yanan Zhai, Yang Zhang, Shuaiming Zhu, Gang Liu* and Yongsheng Che*

***Correspondence:** Yongsheng Che: cheys@im.ac.cn; Gang Liu: liug@im.ac.cn.

1.1 Supplementary Figures

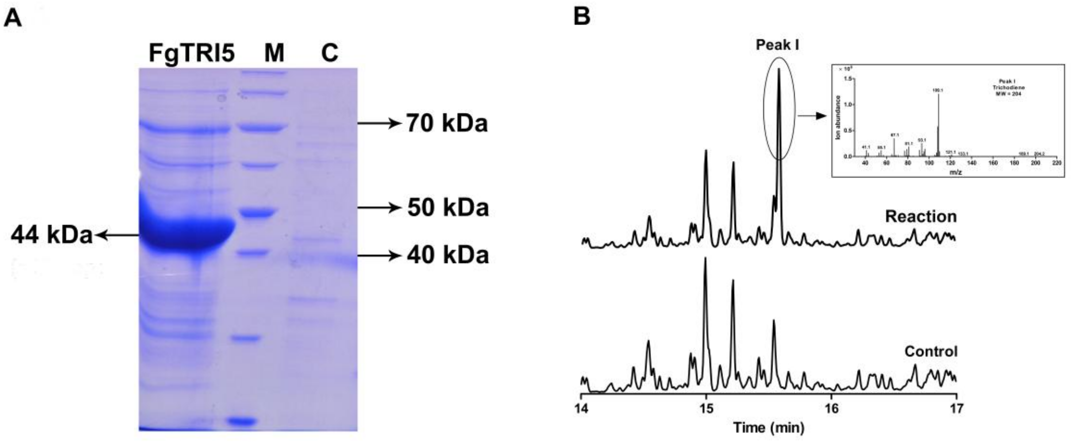

Supplementary Figure 1. SDS-PAGE result of purified recombinant trichodiene synthase FgTRI5 from *E. coli* BL21 (DE3) and GC-MS analyses of *in vitro* enzymatic assay products. (**A**) SDS-PAGE of purified and concentrated FgTRI5 (ca 44 kDa). M: PageRuler^{TM} Broad Range Unstained Protein Ladder (Thermo Fisher Scientific; Waltham, MA, USA); C: The negative control. (**B**) GC-MS analyses of the organic phases (*n*-pentanes) of the reaction mixture and the control in enzymatic assay (top: the total ion chromatogram of trichodiene with mass spectrum inserted; bottom: the control). Peak I represented the newly observed product, showing the same molecular mass and fragments as trichodiene.

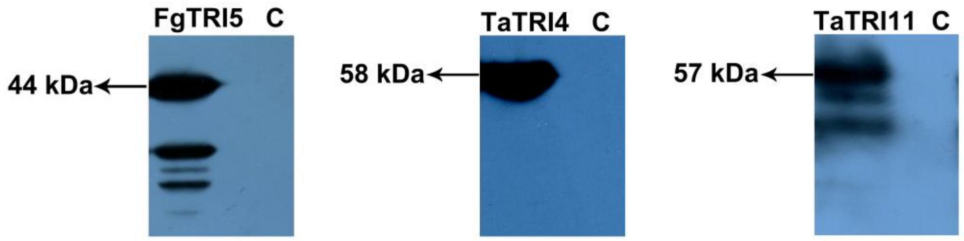

Supplementary Figure 2. Western blot results for the expression of *FgTRI5* in TD1 (left; *ca* 44 kDa), *TaTRI4* in TD5 (middle; 58 kDa), and *TaTRI11* (right; 57 kDa) in TD5. C: The negative control.

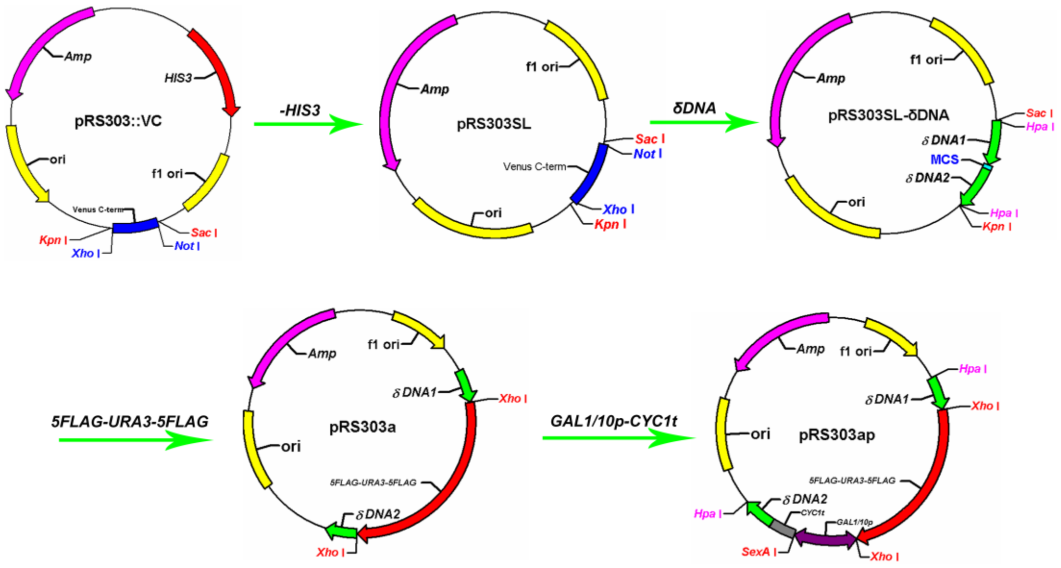

Supplementary Figure 3. Construction of the integration plasmid pRS303ap.

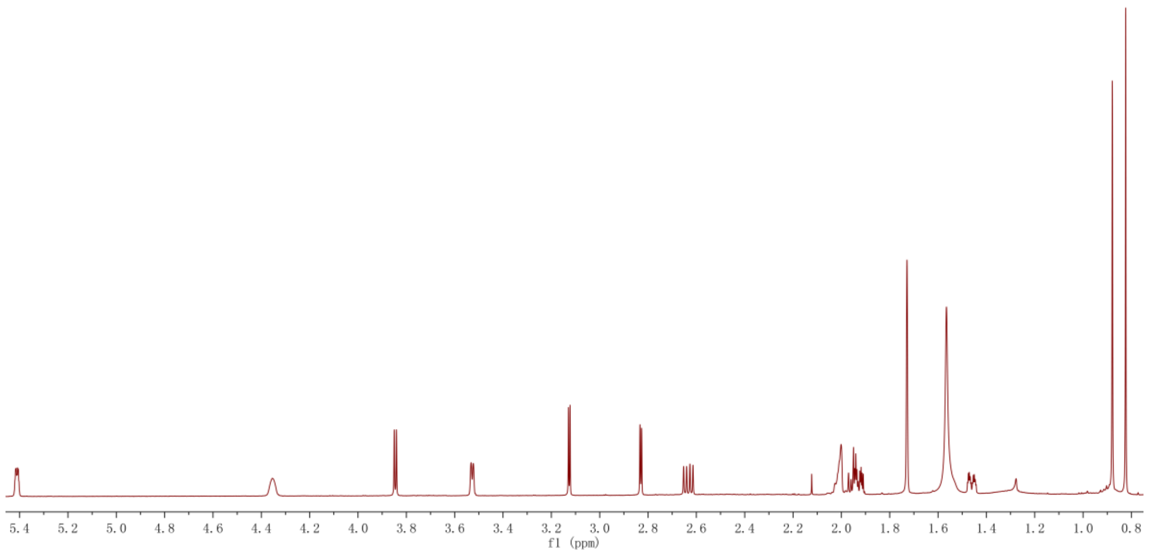

Supplementary Figure 4. ^{1}H-NMR spectrum of authentic trichodermol prepared by hydrolysis of trichodermate A using deuterated chloroform ($CDCl_3$) as the solvent.

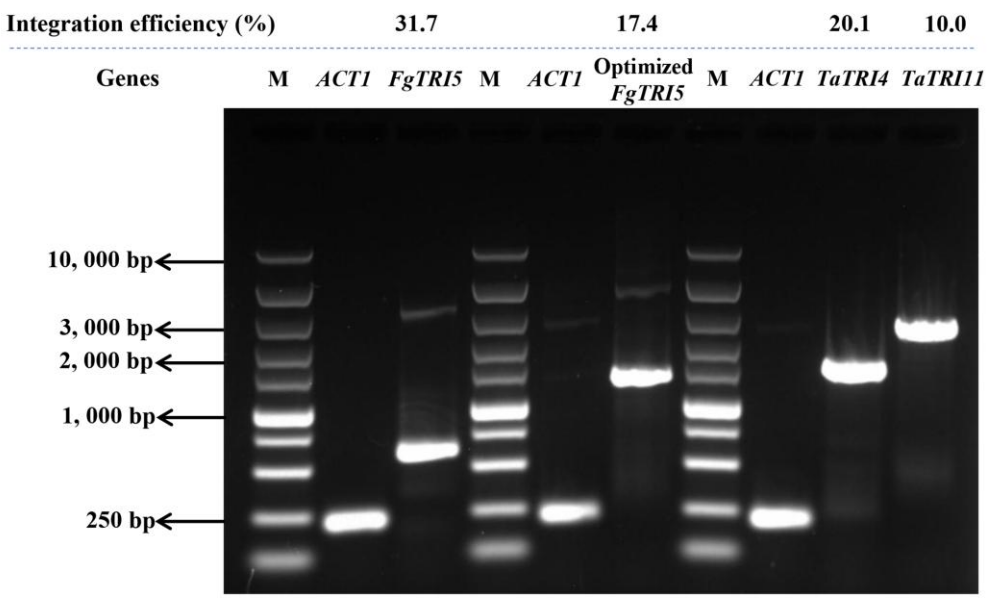

Supplementary Figure 5. Integration efficiencies for *FgTRI5* in TD3, codon-optimized *FgTRI5* in TD4, and *TaTRI4* and *TaTRI11* in TD5, based on Integrated Density Value (IDV) analysis. M: Super DNA Marker (CWBIO, Beijing, China).

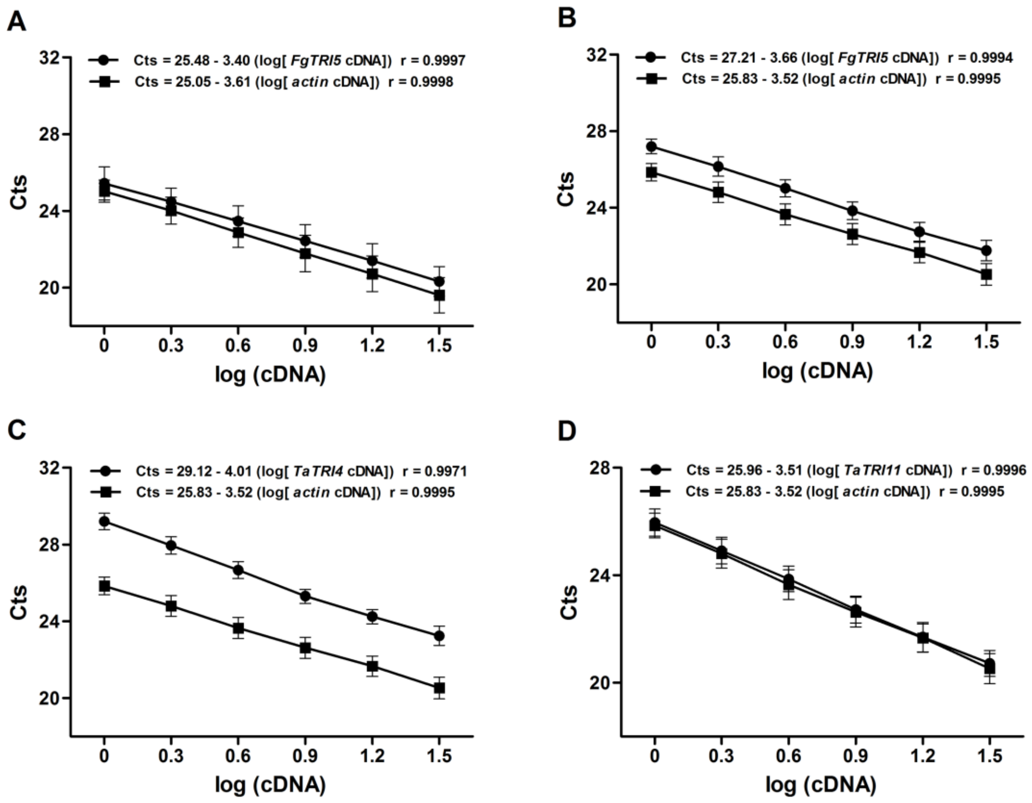

Supplementary Figure 6. Calibration curves from the genomic DNAs of transformants TD3–TD5 used to calculate the copies of *FgTRI5* (**A**), the codon-optimized *FgTRI5* (**B**), and *TaTRI4* (**C**) and *TaTRI11* (**D**) integrated into the genomes of TD3, TD4, and TD5, respectively.

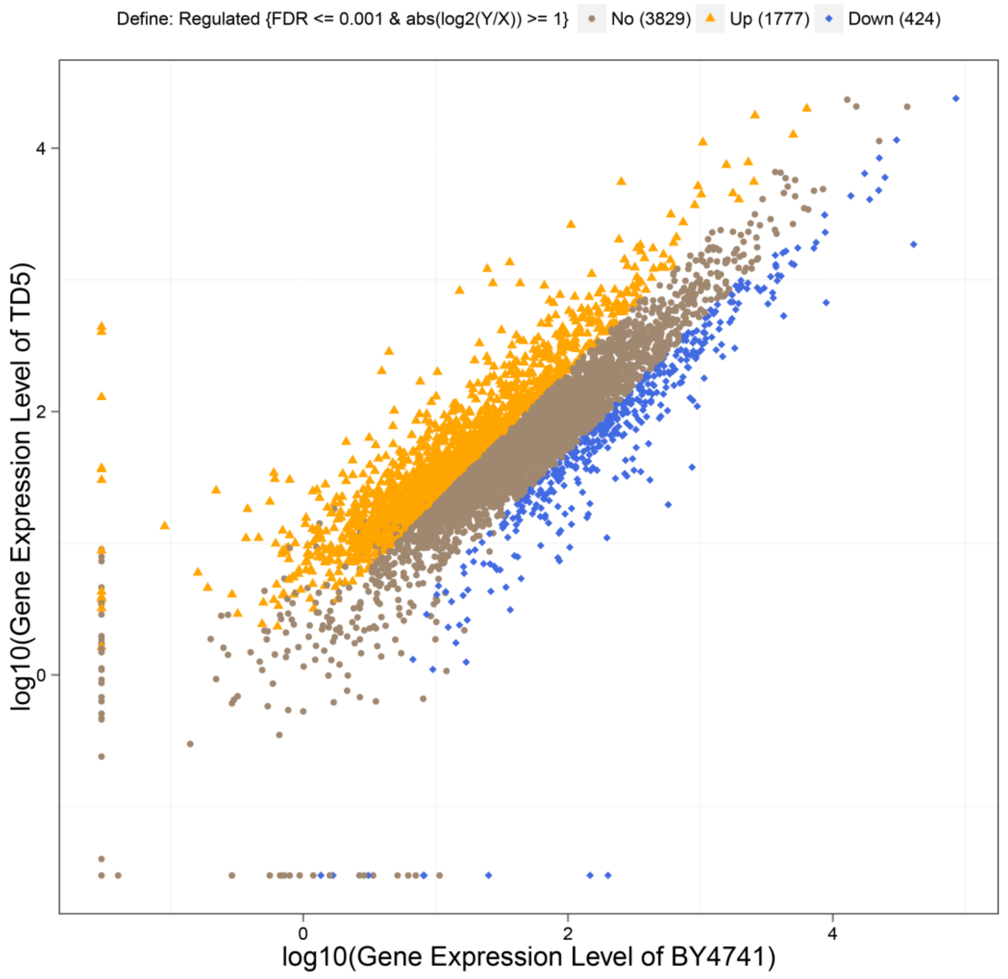

Supplementary Figure 7. Scatter plot for differential gene expressions (DEGs) based on the results from RNA-Seq analysis of TD5 in comparison with BY4741. Blue, orange, and brown points represented the downregulated, upregulated, and unaffected genes, respectively.

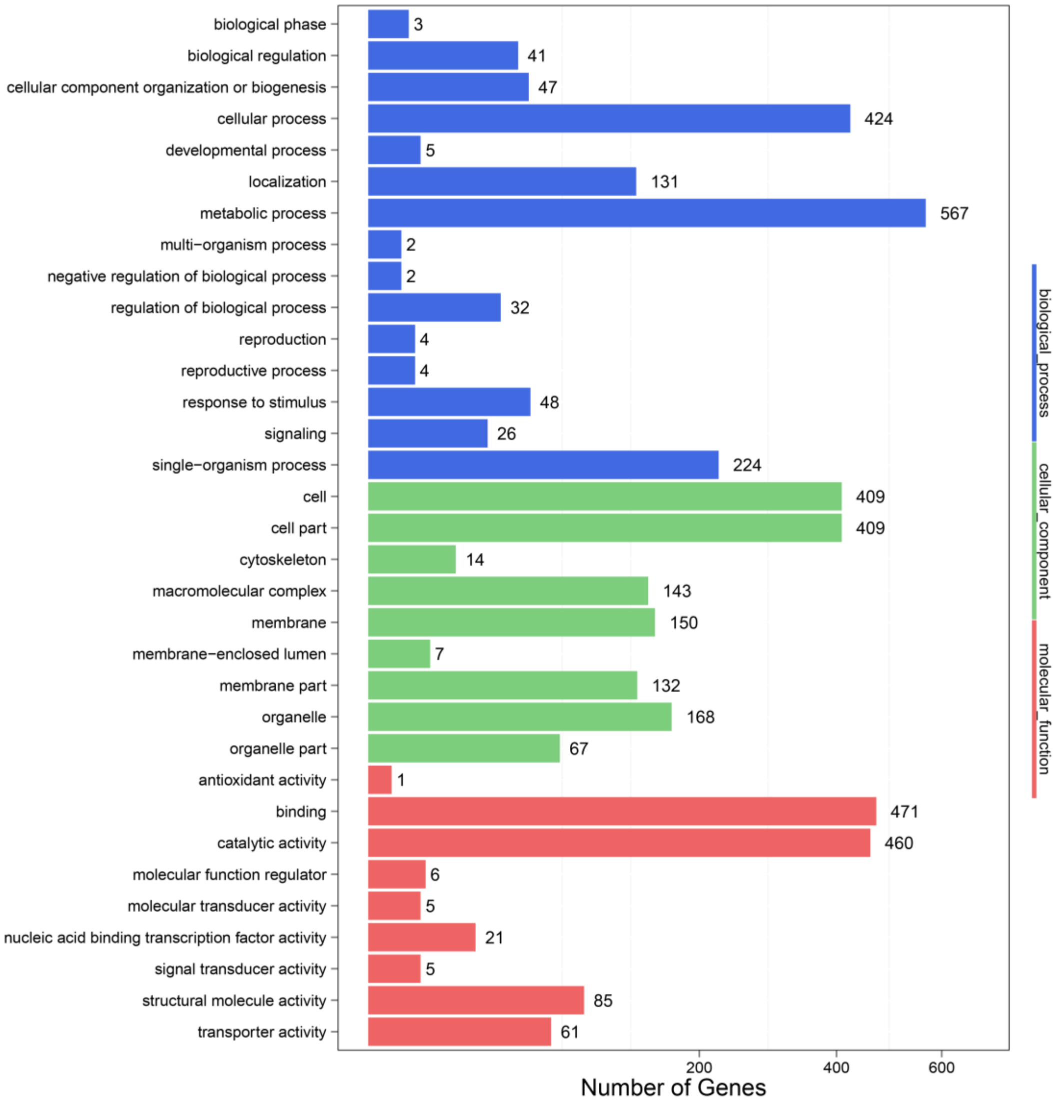

Supplementary Figure 8. Gene Ontology Consortium (GO) function classification of DEGs.

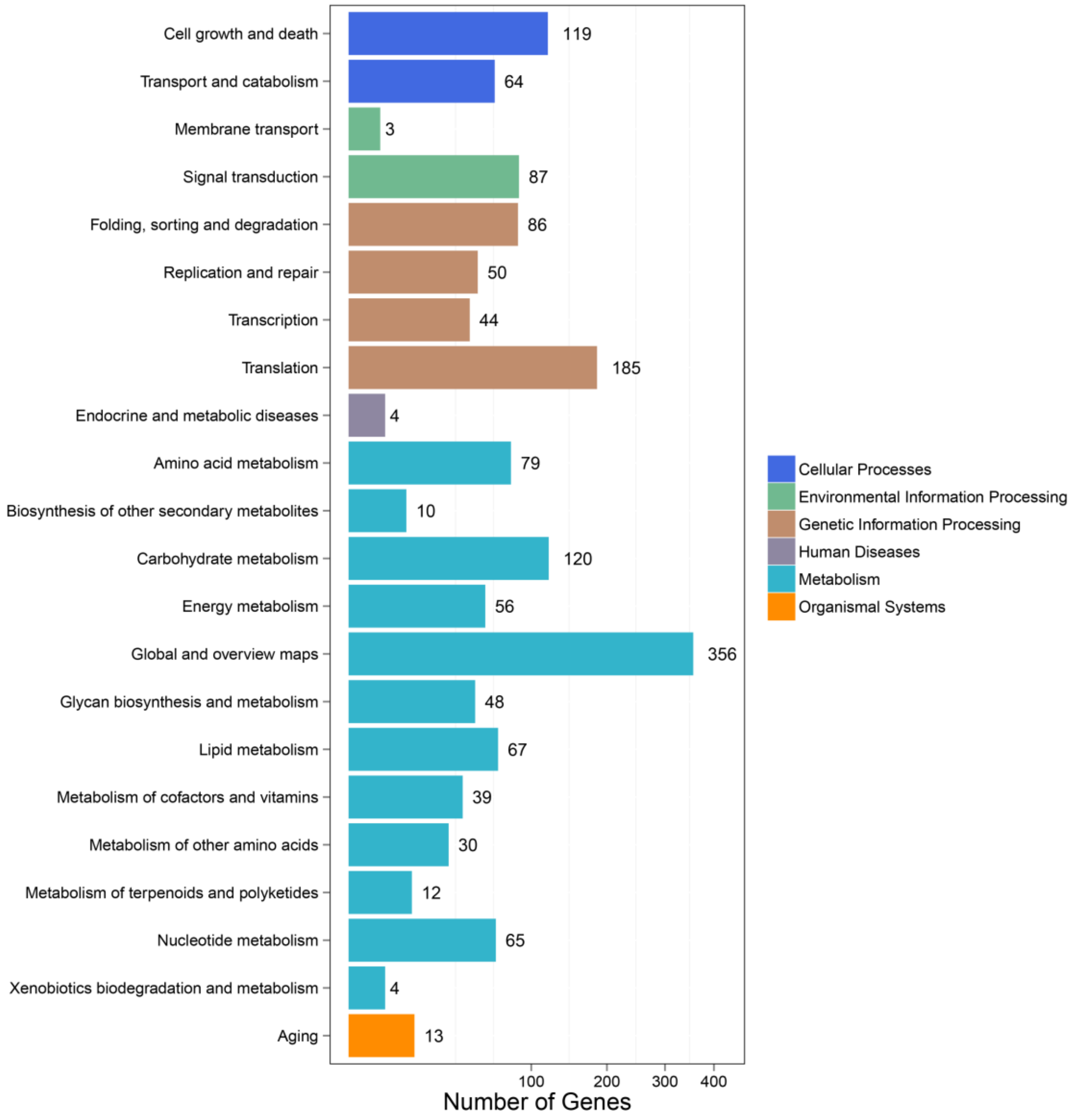

Supplementary Figure 9. Kyoto Encyclopedia of Genes and Genomes (KEGG) function classification of DEGs.

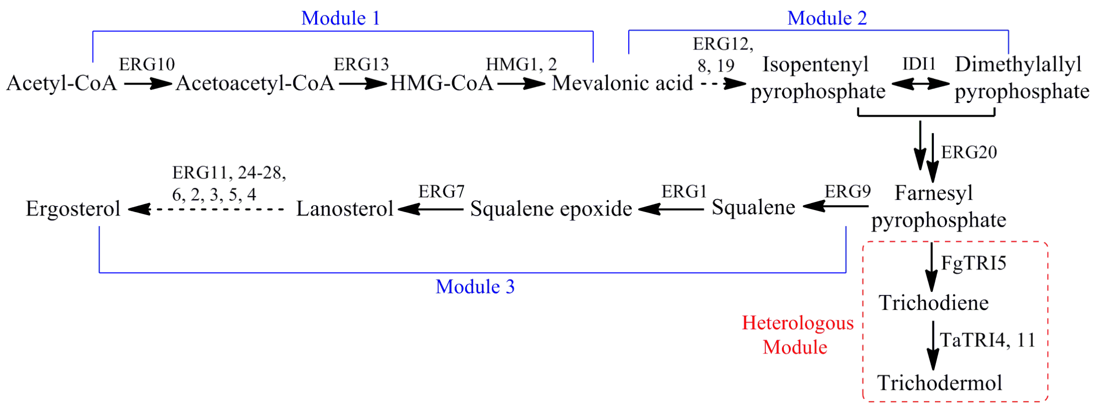

Supplementary Figure 10. Three modules involved in the biosynthetic pathway of ergosterol in *S. cerevisiae*. CoA: coenzyme A; HMG-CoA: 3-hydroxy-3-methylglutaryl-coenzyme A.

1.2 Supplementary Tables

Supplementary Table 1 Oligonucleotides used in this study

Primers	Sequence (5’-3’)[a]
FgTRI5-30a-F	GCGGCCGCATGGAAAACTTTCCCACC
FgTRI5-30a-R	GCGGCCGCCTCCACTAGCTCAATTGAAC
FgTRI5-pESC-F	GGATCCATGGAAAACTTTCCCACCG
FgTRI5-pESC-R	GGTACCTTACTCCACTAGCTCAATTGAACT
FgTRI5-303ap-F	AAACCCTCCGGAACCWGGTATGGAAAACTTTCCCACCG
FgTRI5-303ap-R	GAGCGGATACCWGGTTTAATGGTGATGGTGATGATGCTCCACTAGCTCAATTGAACT
pRS303-L-F	CTGAGAGTGCACCATAATTCCGGATCATATGCGGTGTG
pRS303-L-R	GACGAAAGGGCCTCGTGA
pRS303-S-F	TATCACGAGGCCCTTTCGTCTCGCGCGTTTCGGTGATG
pRS303-S-R	GAATTATGGTGCACTCTCAGTACAA
δDNA1-F	GAGCTCGTTAACATGAAGCAGGTGTTGTTGTCTGT
δDNA1-R	GGATCCCCGCGGGCGGCCGCCTCGAGGAGGAGAACTTCTAGTATATTC
δDNA2-F	GCGGCCGCCCGCGGGGATCCGAGGATATAGGAATCCTCA
δDNA2-R	GGTACCGTTAACGTGTTGGAATAGAAATCAACTATC
FLAG-URA3-F	TAGAAGTTCTCCTCCTCGAGATGGACTACAAGGACGACGAT
FLAG-URA3-R	CCGCGGGCGGCCGCCTCGAGGAATTCGAGCTCGTTTAAACTA
GAL1-10p-F	GAATTCCTCGAGGCGGCCGCTTTCAAAAATTCTTACTTTTT
GAL1-10p-R	GGGTTTTTTCTCCTTGACG
CYC1t-F	ACGTCAAGGAGAAAAAACCCTCCGGAACCWGGTATCCGCTCTAACCGAAAAGG
CYC1t-R	GGATCCCCGCGGGCGGCCGCCTTCGAGCGTCCCAAAAC
TaTRI4O-F	AGAAAAAACCCCGGATCCATGTTGGATATCAACGCT
TaTRI4O-R	CTTCTGTTCCATGTCGACCAATTTCTTAATAACTTTAAC
TaTRI11O-F	TTTTGAAAATTCGAATTCATGGCTAATGCAATTTCTG
TaTRI11O-R	TTAGTGAGGGTTGAATTCTTTAGAAGCAAATCTATCATGC
rDNA1-F	ATGAGAGTAGCAAACGTAAGTC
rDNA1-R	CTCACTATTTTTTACTGCGG
Ta411-F	TATTTTCCGCTTCCGCTTCCGCAGTAAAAAATAGTGAG

	GAGCGACCTCATGCTATACCT
Ta411-R	CTTCGAGCGTCCCAAAAC
HIS3-F	GTACAGACGCGTGTACGCATGTAACATTATACTGAAA ACCTTGCTTGAGAAGGTTTTGGGACGCTCGAAGAGCT TGGTGAGCGCTAGGA
HIS3-R	ATGATCCGTCGAGTTCAAGAG
rDNA2-F	TTGCTTTTTCTTTTTTTTTCTCTTGAACTCGACGGATCA TGAACTGGGTTACCCGGGG
rDNA2-R	TTTCCTCTAATCAGGTTCCACC
ERG1-F	TCGTTTGTCGGTATGTCT
ERG1-R	GCTGGCAAGTAGGAGTTT
ERG7-F	GGTGGGTTCATACTAGAGGTG
ERG7-R	ACAAGGCTGTTCGCAATA
ERG9-F	GTTTGACCCGTTTGATTG
ERG9-R	GGAGCGTATTGTGACCAG
ERG10-F	TTGGTTCATTCCAGGGTT
ERG10-R	TCATAGCGGATGCACAGA
ERG13-F	GCTGGTTCGGATGCTTTG
ERG13-R	TTCGGCGTCAACTTCTGG
ERG20-F	TCTTTCTACTTGCCTGTCG
ERG20-R	ATGCCTTGTTGATTACCC
HMG1-F	AAGTGGACGGTGATTTGA
HMG1-R	AGGTTTCCTGTTGTGGGT
IDI1-F	TGACGAAAGCGGAGAAAC
IDI1-R	AGACGGAGAATGCACGAT
UPC2-F	ACGATCAAGAAGGAGCAG
UPC2-R	ATGGCGGTAATAGTAAGC
PDR5-F	CTTACAGCGGCTACTCAG
PDR5-R	TTCGCATAACTTTCCCTA
PDR11-F	TATCTGACGGTGGAGCAAAC
PDR11-R	AAATTCTAGGGCGGTGGC
PDR12-F	GCTGGTGCTTTCTTCCAGT
PDR12-R	GGACCCATAAACCACCGA
PDR1-F	GGTCATTATCCGTGTCTG
PDR1-R	ACTGAAGTGGGTATTTGC
PDR3-F	GAAGACCCGCACATCACT
PDR3-R	TATGGCAACCAAACTCGA
YOR1-F	AGAGCCTTTCCTATTTCC
YOR1-R	TCATTTCGGGCTTTCTAT
SNQ2-F	GTCGCCAAACCAGACTTA
SNQ2-R	TTGTCCACCCTTCCTCAA
STB5-F	GAACAACATCGTGGGTCA
STB5-R	TTCGTCCTGAGAAACCATAC
RAM1-F	TCCCTTTGCGATAACATA

RAM1-R	CCACTTCTCCGACTTCTA
STE14-F	CAGACATCAGACGCAATC
STE14-R	GACAGGGCAATAATAAAGAG
ACT1-F	CGACGCTCCTCGTGCTGT
ACT1-R	TGGGGCAACTCTCAATTCGT
TRI5-RT-F	CATGGCGGATCTATCTATTC
TRI5-RT-R	TGGGAAAGTGCTCGTTGA
TRI5O-RT-F	TACGCATACAATAAGGCT
TRI5O-RT-R	ATTGACAAATCAGCCATA
TaTRI4-RT-F	TTTTCTTGTTTGCTGGTA
TaTRI4-RT-R	GTATGTTCCTTGTATTGC
TaTRI11-RT-F	GTCTTACATCCCAGGTCC
TaTRI11-RT-R	GCATGATCGATTTCGTTA
TRI5-δDNA2-F	ATACAGAGGACGCCAAGAAG
TRI5-δDNA2-R	TGTTAGAAGATGACGCAAATGA
TRI5O-δDNA2-F	CAAAGGAATGTATGGCTGAT
TRI5O-δDNA2-R	TATACGGTGTTAGAAGATGACG
rDNA1-Ta11-F	GTCCGCATTGGGATGTTAC
rDNA1-Ta11-R	TGGAAGCTGGTGCTGCAT
Ta4-rDNA2-F	CAGATTTCCATTTCGTTGT
Ta4-rDNA2-R	CTCTGTCGCTCTGCCTTA

a The underlined nucleotide sequences indicate restriction enzyme sites

Supplementary Table 2 The codon-optimized sequences of *FgTRI5*, *TaTRI4*, and *TaTRI11*

Proteins	Encoding sequences
FgTRI5 from *F. graminearum* (As_FgTRI5)	ATGGAAAACTTCCCAACTGAATACTTTTTGAACACA<br>TCTGTTAGATTGTTGGAATACATCAGATACAGAGATT<br>CAAACTACACTAGAGAAGAAAGAATCGAAAATTTG<br>CATTACGCATACAATAAGGCTGCACATCATTTTGCTC<br>AACCAAGACAACAACAAATGTTGAAGGTTGATCCA<br>AAGAGATTGCAAGCATCTTTGCAAACAATCGTTGGT<br>ATGGTTGTTTACTCTTGGGCAAAAGTTTCAAAGGAA<br>TGTATGGCTGATTTGTCAATCCATTACACTTACACAT<br>TGGTTTTAGATGATTCTTCAGATGATCCACATCCAGC<br>TATGTTGAATTACTTTGATGATTTGCAAGCTGGTAGA<br>GAACAAGCACATCCATGGTGGGCTTTGGTTAACGAA<br>CATTTCCCAAACGTTTTGAGACATTTCGGTCCATTCT<br>GTTCTTTGAATTTGATCAGATCAACTATGGATTTCTT<br>TGAAGGTTGTTGGATCGAACAATACAACTTCGGTGG<br>TTTCCCAGGTTCTGATGATTACCCACAATTTTTGAGA<br>AGAATGAATGGTTTAGGTCATTGTGTTGGTGCTTCTT<br>TGTGGCCAAAGGATTTGTTCGATGAAAGAAAGCATT<br>TCTTGGAAATTACATCAGCTGTTGCACAAATGGAAA<br>ATTGGATGGTTTGGGTTAACGATTTGATGTCTTTCTA<br>CAAGGAATTTGATGATGAAAGAGATCAAATCTCATT<br>GGTTAAAAATTTCGTTACTTGTCATGAAATCACATTG<br>GATGAAGCATTGGAAAAGTTGACTCAAGAAACATT<br>GCATTCTTCAAAGCAAATGGTTGCTGTTTTCTCTGAT<br>AAAGATCCACAAGTTATGGATACTATCGAATGTTTCA<br>TGCATGGTTACGTTACATGGCATTTGTGTGATGCAAG<br>ATACAGATTGCATGAAATCTATGAAAAAGTTAAAGA<br>TCAAGATACTGAAGATGCTAAGAAATTCTGTAAGTT<br>TTTCGAACAAGCTGCAAATGTTGGTGCTGTTGCACC<br>ATCAGAATGGGCTTACCCACAAGTTGCTCAATTAGC<br>AAACGTTAGAGCAAAGGATGATGTTAAGGAAGCTC<br>AAAAGCCAATCTTGTCTTCAATTGAATTAGTTGAATA<br>A

TaTRI4 from *T. arundinaceum* (As_TaTRI4)

ATGTTGGATATCAACGCTTTGAAGGAAATCCCACCA
GCTACTGCAGTTGGTGCTGCAGTTGCTGTTGGTGCA
TTGTATTTCTTTTGTCAATGTTTCTACAATTTGTACTT
ACATCCATTAAGAAAAATTCCAGGTCCAAAATTGGC
TGCAATTGGTCCATATTTGGAATTCTACCATGAAGTT
TTGAGAGATGGTCAATATTTGTGGGAAATTGAAAAG
ATGCATCAAAAATACGGTCCAATTGTTAGAGTTAAC
GCTAGAGAAGTTCATGTTAAGGATACTTCTTACTACA
ACACTATCTATACAGCTGGTGCAAGAAAAACTAATA
AGGATCCAGCAACAGTTGGTGCTTTTGATGTTCCAA
CTGCAACAGCTGCAACAGTTGATCATGATTTGCATA
GAGCTAGAAGAGGTTATTTGAACCCATACTTCTCTA
AAAGAGCTGTTGCAGGTTTGGAACCAACTATCCATG
AAAGAATCACAAAGTTGTTATCAAGATTCGATCAAC
ATAGAAAGGATGATCAAGTTTTGTCTTTAGATGGTG
CTTTTTCAGCATTAACTGCTGATGTTATCACAGCAAG
ATTCTACGGTGAACATAAGGATTACTTGGATGTTCCA
GATTTCCATTTCGTTGTTAGAGATGGTTTCCAAGGTT
TGTCTAGAGTTTACCATTTGGGTAGATTTTTACCATC
AGTTGTTGGTGCTTTGAAAGGTTTGCCAAAGTTCTT
GATCAGAATCATCTTCCCACCAATCGCAGAATTGTT
GACTATGAGAGAAGAAATCGAAGCTGGTGGTATCGA
TGAATTCACTAAGTCTAAATCTTCAGGTATTAAATCT
TCAGTTTTGGTTGGTGCTTTATCAGATCCACATATTC
CACCACAAGAAAGAACTGTTGCAAGAATGTTGGAT
GAAGGTACTGTTTTCTTGTTTGCTGGTACAGAAACT
ACATCTAGAACTTTGGGTATCACAATGTTCTATTTGT
TATCAAATCCAGATATTTTGAATAAGTTAAGAGAAGA
ATTGAAATCTTTACCACCATCAGATGATAACATGCAT
TCTTTGGGTCAATTGGAAAATTTGCCATACTTAACAG
GTGTTGTTCATGAAGGTTTGAGATTATCTTTTGGTCC
AATTTCAAGATCTTCAAGAGTTGCAACTCATGAAGC
TTTGCAATACAAGGAACATACTATTCCAGCAGGTAC
ACCAGTTTCTCAATCAACATACTTCGTTCATACTGAT
ACAGAAATCTTCCCAGATCCATGGGAATTCAAACCA
GAAAGATGGATTAAAGCTGCAGAAGATGGTGTTGCT
TTGAAGAAATACATCACTAACTTCTCTCAAGGTTCA
AGACAATGTATTGGTTACTCTATGTCATTCGCAGAAA
TGTTCTTGACATTGTCTAGAATCATCCCAGCTTTCGA
TTTGGAATTATACGATACTACAAAAGCAGATATTGAT
ATGACTCATGCTAGAATTGTTGGTTACCCAAAGAAA
GTTCCAGGTAAAACAGAATCATTGGGTGAATTAAGA
GTTAAAGTTATTAAGAAATTGTAA

TaTRI11 from *T. arundinaceum* (As_TaTRI11)	ATGGCTAATGCAATTTCTGTTGGTGTTGCAGTTCAAT<br>TGGTTTTGACTGTTTTGTTGGCTTCAATCCCATTGAG<br>AGTTATTTGGAATTTGTTTTTCCATCCATTGTCTTACA<br>TCCCAGGTCCAAGATTGTGGATCGCTTTCCCAATTTT<br>TAGACAAATCGCATCTATTAGAGGTGTTTTCGATGCT<br>CAAATGTGTGAATACCATAGAAAGTACGGTAACGCA<br>GTTAGATTTTCTCCAAACGAAGTTTCTTTTATTACTG<br>AACAAGCTTGGAGAGATATCTATGATCATAGACCAA<br>ACCAATTGGAAAGATTCATTTTGTCAACTACAAGAA<br>GACCAGATATCTTCGATGCTAACGAAATCGATCATGC<br>AAGATATAGAAAGGCTATGTTACCAGCATTTTCTCCA<br>AAAGGTTTGCAAGAACAAGAACCAATTGTTAGAGG<br>TTACATCGATACTTTTATTGAAAGATTGAGAGAAGTT<br>TCTGCAACTGGTGAATCAACAGATATGGTTAAGTGG<br>TACAACTTCACTACATTCGATATTATTGGTGACTTGG<br>CTTTTGGTGAATCATTTGGTGGTTTGAGAAATAGAG<br>AATACCATTTTACTATCTCTTTTACATTCGAAGCTTTT<br>AAATTGTTATCATATTTGGAAGCTGGTGCTGCATACC<br>CATTGTTGTTGAAGATCTTGATGGCTTTTACTCCACA<br>ATCTTTGATTGAAGCAAGAGATAAGAAAGAAGAAC<br>ATGCTGAAACTACAGTTAGAAAGAGATTGGATAACA<br>GAGCTTTGCATGGTCGTGGTGACTTCATGGATTATTT<br>GTTGAGAAACAGAGGTGAAAAGCAAGGTTTGAACG<br>ATAAGGAATTGGTTGCTAACGCATCTACTTTGATCAC<br>AGCTGGTTCAGAAACTACAGCAACTATCTTGTCAGG<br>TATCACATACTGGTTGTTGCAAACTCCAAACGTTTTG<br>CAAAAGGTTACTGAAGAAGTTAGATCTGCTTTCCAA<br>TCAGAAGCAGATATCACTTTTACATCTGCTACATCAC<br>AATTACCATATATGTTGGCTTGTTTTCAAGAAGCTTT<br>TAGACATTACCCACCAGTTCCAACTGGTATGCCAAG<br>AGTTACTCCATCTCATGGTATCACAAAGATCTCTGGT<br>TACGATATCTCACCAAACACAAAAGTTTCTGTTCATC<br>AATTAGCTGCATACTCACATCCAGATAACTTCCATAG<br>ACCAAGAGAATTCGTTCCAGAAAGATGGTTGCCAG<br>ATGCTAAAACTAATCCATCTTCACCATGGTATAATGAT<br>AGAAGAGAAACAGTTCAACCTTTTAATGTTGGTCCA<br>AGAAATTGTGTTGGTAGAAATTTGGCAGAACAAGA<br>AATCAGAGTTATGTTGGCTAGAGTTTTGTGGAACTT<br>CGATTTGGAATTGGCTCCAGAATCTAAAAATTGGAC<br>TGATCAAAAGACTCATTTCTTGTGGGAAAAAGGTGC<br>ATTGATGTGTAAGTTGCATGATAGATTTGCTTCTAAA<br>TAA

Supplementary Table 3 Genes related to terpenoid metabolism and ABC transporters in TD5

Gene	Gene ID	Characterization
Terpenoids metabolism		
RAM1	851468	Subunit of the CAAX farnesyltransferase
STE14	852019	Farnesyl cysteine-carboxyl methyltransferase
MVD1	855779	Mevalonate pyrophosphate decarboxylase
ABC transporters		
PDR5	854324	ABC transporter
PDR11	854802	ABC transporter
PDR12	856049	ABC transporter
YOR1	853198	ABC transporter
SNQ2	851574	ABC transporter
PDR1	852871	Drug-responsive transcription factor
PDR3	852278	Drug-responsive transcription factor
STB5	856583	Transcription factor
